# Replication Study of *ENPP1* Variants and Haplotypes Associated with Severe Obesity in a Greek Adult Population

**DOI:** 10.1007/s10528-026-11413-2

**Published:** 2026-06-20

**Authors:** Konstantinos Rouskas, David Meyre, Stephane Lobbens, Dimitrios Papazoglou, Anastasia Kouvatsi, Philippe Froguel

**Affiliations:** 1 Institute of Applied Biosciences, Centre for Research & Technology Hellas, 54124 Thessaloniki, Greece; 2 INSERM U1256, Nutrition, Genetics, and Environmental Risk Exposure (NGERE), University of Lorraine, 54500 Nancy, France; 3 Division of Biochemistry, Molecular Biology, and Nutrition, Department of Molecular Medicine, University Hospital of Nancy, 54500 Nancy, France; 4 Inserm U1283, CNRS UMR 8199, European Genomic Institute for Diabetes, Institut Pasteur de Lille, Lille, France; 5 Lille University Hospital, University of Lille, Lille, France; 6 Second University Department of Internal Medicine, University Hospital of Alexandroupolis, Democritus University of Thrace, Alexandroupoli, Greece; 7 Department of Genetics, Development and Molecular Biology, School of Biology, Aristotle University of Thessaloniki, Thessaloniki, Greece; 8 Department of Metabolism, Imperial College London, Hammersmith Hospital, London, UK

**Keywords:** Obesity, *ENPP1*, Genetic variants, Replication study, Haplotype, Greek population

## Abstract

The *ENPP1* (Ectonucleotide pyrophosphatase/phosphodiesterase 1) gene, encoding a protein that negatively modulates insulin receptor activation, remains relatively understudied in obesity genetics. K121Q (rs1044498; chr6:132,172,368: A > C) and other *ENPP1* genetic variants (rs1799774; chr6:132,203,471: insT> delT and rs7754561; chr6:132,212,694: A > G), as well as the risk haplotype Q–delT–G have been investigated for their association with obesity risk mostly in populations from Central and Northern Europe, yielding controversial findings. Given the significance of reproducibility in genetic association studies and the scarcity of data from Southern Europe, we performed a case-control replication study to test the hypothesis that *ENPP1* variants and haplotypes are associated with severe obesity in the underexplored and high-risk Greek population. A total of 979 Greek individuals (mean age: 47.6 ± 12.5years old), including 510 adults with severe obesity (BMI≥35Kg/m^2^) and 469 normal-weight (18.5 < BMI<25Kg/m^2^) controls, were studied. Three *ENPP1* variants (rs1044498, rs1799774 and rs7754561) were genotyped using the TaqMan allelic discrimination assay. Logistic regression was applied to detect association of variants with obesity. Linkage disequilibrium among the three variants was assessed, and haplotype frequencies were compared between groups. The three variants did not exhibit any association with adult severe obesity. The three variants were in partial linkage disequilibrium (r^2^ ≤ 0.43). *ENPP1* haplotype distribution differed significantly between normal-weight individuals and those with adult severe obesity (likelihood ratio x^2^ = 13.38, *P* = 0.020), with the Q–delT–G haplotype found to be present exclusively in individuals with adult severe obesity (frequency 3.3%) and to be associated with adult severe obesity (x^2^ = 5.66, *P* = 0.017; reference haplotype K–InsT–A, consisting of the three major alleles of the three variants). Our replication findings contribute novel evidence regarding the genetic architecture of adult severe obesity in a Southern-European population, however larger studies are necessary to validate these results.

**Supplementary Information:**

The online version contains supplementary material available at https://doi.org/10.1007/s10528-026-11413-2.

## Introduction

Obesity is a complex multifactorial disease that contributes to increased risk of diabetes, cardiovascular disease and several cancers and to reduced life expectancy (Bluher 2025). Obesity prevalence rises persistently during the last decades (World Health Organization 2024), with over 50% of adults predicted to be overweight or obese by 2050 (Collaborators 2025). Particularly in Europe, almost one fourth of people are living with obesity (Dicker et al. 2025), with South-European countries including Greece displaying among the highest obesity rates in adults (Authority 2021).

Polygenic forms of obesity have a strong genetic basis with over 1,000 loci found to associate with obesity risk through genome-wide association studies (GWAS) (Bonnefond et al. 2026). Among the least studied genes associated with obesity is *ENPP1* (Ectonucleotide pyrophosphatase/phosphodiesterase 1). ENPP1, also known as plasma cell membrane glycoprotein 1 (PC-1), is a membrane glycoprotein that impairs insulin signaling by binding to the insulin receptor (Maddux and Goldfine 2000), has been implicated as a negative regulator of innate immune system-related pathways and is considered as a promising target for cancer immunotherapy (Wang et al. 2023). A specific variant in exon 4 of *ENPP1* gene (K121Q-rs1044498) has been widely studied and linked to inhibitory activity in the insulin signalling (Costanzo et al. 2001). The Q121 allele has been found to associate with insulin resistance (Frittitta et al. 2001; Meyre et al. 2007; Di Paola et al. 2011; Arianti et al. 2021; Li and Liu 2025) and with increased risk of adult obesity and type 2 diabetes in populations of European ancestry (McAteer et al. 2008; Wang et al. 2011). Meyre et al. (Meyre et al. 2005) described a three-variant at-risk haplotype in the *ENPP1* gene (formed by the three minor alleles of rs1044498 Q-allele, rs1799774 delT allele, and rs7754561 G-allele) that was associated with increased risk of childhood and adult obesity and type 2 diabetes. However, associations of the above-mentioned *ENPP1* gene variants and haplotypes with obesity risk remain controversial and were mostly explored in Central-Northern European and Asian populations (Bottcher et al. 2006; Lyon et al. 2006; Weedon et al. 2006; Meyre et al. 2007), highlighting the need for reproducibility (Li and Meyre 2013) in other understudied populations with different lifestyles, ethnic and environmental backgrounds.

To address this knowledge gap and to continue our previous work on assessing the genetic basis of adult obesity in the understudied and high-risk Southern-European Greek population (Kapantais et al. 2006; Rouskas et al. 2008, 2012a, b), we conducted a case-control replication study among Greek adults. The aim of the study was to investigate the association between severe obesity and the three *ENPP1* variants rs1044498, rs1799774 and rs7754561, as well as the risk haplotype Q–delT–G in the Greek adult population. Our findings suggest a potential cumulative role of *ENPP1* gene variants on increased susceptibility to adult severe obesity in the Greek population, representing the first, to our knowledge, study that explores association of *ENPP1* gene variants and haplotypes with adult severe obesity in South-European populations.

## Materials and Methods

### Subjects and Phenotypes

All individuals provided written informed consent, and the protocol was approved by the local ethics committee of CERTH in Thessaloniki, Greece (approval number ETH.COM-60/18-05-2021), based on the Helsinki declaration. Nine hundred seventy-nine individuals were enrolled to participate in this case-control replication study. Participants’ characteristics, recruitment and inclusion criteria were described previously (Rouskas et al. 2012a). Briefly, 469 participants were classified as normal-weight control subjects (18.5 < BMI<25Kg/m^2^, mean age: 46.89 ± 12.04years, mean BMI: 23.71 ± 0.95Kg/m^2^) and 510 subjects were classified as individuals with severe obesity (BMI≥35Kg/m^2^, mean age: 47.79 ± 12.51years, mean BMI: 37.59 ± 6.47Kg/m^2^). Inclusion criteria for normal-weight individuals were 18.5 < BMI<25Kg/m^2^ at the examination time, absence of a history of obesity in the subject and among first-degree relatives, absence of diabetes, hypertension, and medical treatment. All individuals self-reported as having a White-European Greek origin and were recruited simultaneously. Matching of sex distribution among groups was not performed at the time of recruitment.

Regarding demographic, anthropometric and blood pressure parameters, all data were collected by a trained nurse practitioner at the collaborating hospitals during participants’ clinic visits. Biochemical tests for glycemic and lipid traits were conducted on fasting blood samples collected from the participants on the morning of their hospital visit, using automated instrumentation in the hospital’s routine clinical examination check-ups.

### Variants Selection and Genotyping

We selected the following variants for genotyping: rs1044498 (chr6: 132,172,368; A > C or K121Q, where C-allele corresponds to Q), rs1799774 (chr6: 132,203,471; insT> delT) and rs7754561 (chr6:132,212,694; A > G in the 3΄ untranslated region). These variants were selected based on their prior association with adult obesity in a linkage analysis in European populations (Meyre et al. 2005). Genotyping was performed using the TaqMan allelic discrimination assay (Applied Biosystems, Foster City, CA) on an ABI7900 system, following the manufacturing protocol (Applied Biosystems). The PCR primers and TaqMan probes were designed by Primer Express and optimized according to the manufacturer’s protocol. Primer sequences are available upon request. Quality control (QC) criteria for variants included a genotyping call rate > 98% and absence of deviation from Hardy-Weinberg Equilibrium (HWE) (Wigginton et al. 2005) (*P* > 0.05).

### Statistical Analysis

Descriptive comparisons between normal-weight individuals and individuals with adult severe obesity were performed using the independent samples T-test (continuous outcomes) and x^2^ test (binary outcomes). Genotypes were checked for the conformance of HWE using the online calculator deFinetti (https://gaow.github.io/genetic-analysis-software/f/finetti/). A de Finetti diagram is used in population genetics to graph genotype frequencies for populations with biallelic variants (Cannings and Edwards 1968). Statistical power calculations were performed with QUANTO software (https://keck.usc.edu/biostatistics/software/) (Gauderman 2002), using the current prevalence of obesity in Greece (Kapantais et al. 2006), the odds ratios (ORs) previously reported in the literature (Meyre et al. 2005), and an α-level of 0.05. Logistic regression models were used to assess the association between variants and binary obesity status. Comparisons were carried out between individuals with adult severe obesity (BMI≥35Kg/m^2^) and normal-weight (BMI<25Kg/m^2^) individuals. Genotypes were coded as 0, 1, and 2, depending on the number of copies of the risk alleles. The risk alleles were defined as those associated with increased obesity risk in previous studies (Meyre et al. 2005). ORs and *P*-values, all adjusted for age and sex, were tested under three genetic modes of inheritance (additive, dominant and recessive). Linkage disequilibrium among the three investigated variants was assessed by estimating pair-wise r^2^ values from genotypes using the program THESIAS (Tregouet and Garelle 2007). Haplotype frequencies were determined and compared between cases and controls using the subprogram Cocaphase of UNPHASED software (Dudbridge 2008). The likelihood ratio x^2^ and *P* value were calculated to compare the global distribution of haplotypes in cases and controls. For consistency with the Meyre et al. (2005), x^2^ and *P* values are presented for each haplotype relative to the most common haplotype. SPSS 14.1 (SPSS, Chicago, IL) was used for general statistical analyses. Two-sided *P* values < 0.05 were considered significant.

## Results

As shown in Table 1, the two groups showed significant differences in all metabolic traits. Anthropometric measures such as BMI and waist circumference, and characteristics related to lipid and glucose homeostasis showed significantly higher values in the group of individuals with adult severe obesity than in their normal weight counterparts. A significant distortion in the gender ratio was observed between normal-weight (64.6% males) individuals and those with adult severe obesity (25.8% males). Given this difference in gender ratio among the two groups, we calculated waist circumference and WHR separately for males and females within each group. Both waist circumference and WHR were statistically significant (*p* < 0.0001, unpaired t-test) higher in males in both the normal-weight (waist circumference; males: 94.49 ± 10.56 cm vs. females: 88.11 ± 9.53 cm, WHR; males: 0.85 ± 0.04 vs. females: 0.82 ± 0.04) and the severe obesity (waist circumference; males: 120.96 ± 14.04 cm vs. females: 112.49 ± 12.89 cm, WHR; males: 1.01 ± 0.09 vs. females: 0.90 ± 0.08) group, respectively.

**Table 1:** Demographic and clinical characteristics of individuals under study

	Normal-weight individuals (*n* = 469)	Individuals with severe obesity (*n* = 510)	*P*
Age (yrs)^a^	46.89 ± 12.04	47.79 ± 12.51	0.619
Men, % (n)^b^	64.6% (303)	25.8% (132)	< 0.001
Weight (Kg)^a^	71.87 ± 6.3	103.37 ± 20.11	< 0.001
Height (m)^a^	1.74 ± 0.07	1.66 ± 0.09	< 0.001
BMI (Kg/m^2^)^a^	23.71 ± 0.95	37.59 ± 6.47	< 0.001
Waist circumference (cm)^a^	92.08 ± 10.86	114.69 ± 13.66	< 0.001
Hip circumference (cm)^a^	109.75 ± 10.73	124.35 ± 12.87	< 0.001
WHR^a^	0.84 ± 0.05	0.93 ± 0.09	< 0.001
SBP (mm Hg)^a^	125.52 ± 6.87	133.87 ± 14.90	< 0.001
DBP (mm Hg)^a^	83.96 ± 5.15	85.5 ± 10.29	< 0.001
FBG (mg/dl)^a^	90.29 ± 8.59	109.06 ± 34.68	< 0.001
HDL-C (mg/dl)^a^	53.54 ± 5.42	50.43 ± 11.12	< 0.001
LDL-C (mg/dl)^a^	122.65 ± 15.75	133.74 ± 35.37	< 0.001
Triglycerides (mg/dl)^a^	125.52 ± 24.92	145.94 ± 74.06	< 0.001
Total cholesterol (mg/dl)^a^	174.16 ± 17.51	211.3 ± 39.40	< 0.001
IFG/IGT, % (n)^b^	0% (0)	14.4% (67)	< 0.001
T2D, % (n)^b^	0% (0)	22.2% (103)	< 0.001

^a^Data presented as means ± standard deviations

^b^Data presented as percentages (n)

Male/Female ratios and differences in IFG/IGT and type 2 diabetes percentages were analyzed using the x2-test. P-values for quantitative traits were analyzed using an independent samples t-test. BMI: Body Mass Index; WHR: Waist to Hip Ratio; SBP: Systolic Blood Pressure; DBP: Diastolic Blood Pressure; FBG: Fasting Blood Glucose; HDL-C: High Density Lipoprotein-Cholesterol; LDL-C: Low Density Lipoprotein-Cholesterol; IFG: Impaired Fasting Glucose; IGT: Impaired Glucose Tolerance; T2D: Type 2 Diabetes; % IFG/IGT: Percentage of individuals having IFG or IGT

### Association of rs1044498, rs1799774 and rs7754561 with Risk of Adult Severe Obesity

All three variants passed the QC criteria (Table S1), harbouring a genotyping call rate between 98.06 and 99.69% and HWE *P* > 0.2. Table S1 shows genotype counts and frequencies of the three studied variants in the two groups. We found statistical power of 66.8%, 79.7%, and 90.6% to detect the previously reported associations for the variants rs1044498, rs1799774, and rs7754561, respectively (Table S2). We did not detect any association between the three *ENPP1* variants and adult severe obesity status in the Greek population under the three tested genetic models (additive, dominant and recessive) (Table 2). Briefly, odds ratios for rs1044498, rs1799774, and rs7754561 ranged from 1.04 to 1.13, 1.04 to 1.10 and 1.07 to 2.41, respectively, with p-values ranging from 0.162 to 0.796 across all inheritance models.

**Table 2:** Association tests of three *ENPP1* gene variants in Greek adults with severe obesity (BMI≥35Kg/m^2^) compared to normal-weight (BMI<25Kg/m^2^) individuals

Nearest Gene	Variant	Major/Minor allele	RA	RA frequency		Additive		Dominant		Recessive	
				Normal-weight individuals	Individuals with severe obesity	*P*	OR (95% CI)	*P*	OR (95% CI)	*P*	OR (95% CI)
*ENPP1*	rs1044498	A > C	C	0.13	0.14	0.421	1.13 (0.84–1.50)	0.633	1.08 (0.79–1.48)	0.162	2.41 (0.70–8.26)
*ENPP1*	rs1799774	insT> delT	delT	0.29	0.31	0.727	1.04 (0.84–1.29)	0.796	1.04 (0.79–1.36)	0.734	1.09 (0.66–1.80)
*ENPP1*	rs7754561	A > G	G	0.37	0.4	0.501	1.07 (0.88–1.30)	0.489	1.1 (0.83–1.46)	0.712	1.07 (0.73–1.57)

P-values for additive, dominant and recessive models were calculated using logistic regression models adjusted for age and sex. RA: Risk allele; OR: Odds Ratio; CI: Confidence Interval

### Association of the *ENPP1 *Gene Haplotypes with Adult Severe Obesity

The linkage disequilibrium among the three studied variants was low: r^2^ = 0.19 between rs1044498 and rs1799774, r^2^ = 0.12 between rs1044498 and rs7754561, and r^2^ = 0.43 between rs1799774 and rs7754561. Meyre et al. described a three-allele haplotype in the *ENPP1* gene (formed by the three minor alleles of rs1044498 Q-allele, rs1799774 delT allele, and rs7754561 G-allele) that was associated with increased risk of obesity in French adults (Meyre et al. 2005). To estimate the potential effects of combinations of the three variants on the risk of adult severe obesity, haplotype analysis was performed using the three variants’ data from the 979 adult Greek individuals. We identified six haplotypes among the three different variants. Haplotype frequencies for normal-weight individuals and individuals with severe obesity and their statistical association analysis results are shown in Table 3. We found a significantly different distribution of *ENPP1* haplotypes among normal-weight and individuals with severe obesity (*P* = 0.02) and a significant association of the Q–delT–G ‘risk haplotype’ with adult severe obesity (x^2^ = 5.66, *P* = 0.0172, using the more common K-insT-A haplotype as reference). The frequency of the Q–delT–G haplotype was 3.3% in the group of individuals with severe obesity and was absent in the normal-weight group.

**Table 3:** Haplotype analysis in individuals with severe obesity (BMI≥35Kg/m^2^) and normal-weight individuals (BMI<25Kg/m^2^)

Haplotypes	Frequencies		Case-Control test under the allelic model	
	Normal-weight individuals	Individuals with severe obesity	x^2^	*P*
K–insT–A	86.98	86.26	0.04	0.843
K–insT–G	4.73	3.29	0.47	0.492
K–delT–A	1.77	0	3.25	0.071
K–delT–G	5.32	6.59	0.25	0.617
Q–insT–A	1.18	0.55	0.42	0.5191
Q–insT–G	0	0	0	NA
Q–delT–A	0	0	0	NA
Q–delT–G	0	3.29	5.66	0.0172

Haplotypes comprising three variants (rs1044498; K121Q, rs1799774; insT> delT and rs7754561; A > G) are shown

Haplotype frequencies were compared between groups using the Cocaphase subprogram of the UNPHASED software. The likelihood ratio x2 and P value were calculated to compare the global distribution of haplotypes between cases and controls

## Discussion

This study aimed to replicate the association between adult severe obesity and *ENPP1* variants rs1044498, rs1799774 and rs7754561 and of the three-allele haplotype Q–delT–G in an adult Greek population.

We were unable to detect a consistent association between the three studied variants and adult severe obesity in the Greek population. The association of the three studied variants, particularly K121Q, with obesity risk in adults and children has been repeatedly reported in the literature through genetic association studies in populations of European (Meyre et al. 2005; Bottcher et al. 2006; Tanyolac et al. 2008) or Asian (Wan et al. 2006; Arianti et al. 2021) descent. A meta-analysis of 24,322 subjects has also confirmed the association between the Q121 variant and obesity (Wang et al. 2011). Table 4 summarizes association findings in other populations for K121Q variant as well as the Q–delT–G risk haplotype. The lack of association between the three variants and obesity in our adult Greek population sample may arise from various factors, such as the modest sample size of the current study, or reflect the impact of different living environments within the Greek population. These observations are in line with current evidence supporting the need to conduct genetic studies across diverse populations to understand complex traits and diseases (Wojcik et al. 2019), including obesity (Loos 2025). However, when haplotypes were tested, we found that the Q–delT–G haplotype is associated with an elevated risk of severe obesity in Greek adults. The relatively low LD among the three variants is in line with observations in populations from France (Meyre et al. 2005), Italy (Morandi et al. 2009), or Germany (Bottcher et al. 2006) and indicates that these variants may represent independent genetic signals making haplotype analysis more informative; however, interpretation of these findings needs caution and validation in larger studies. The association of Q–delT–G with obesity risk in the Greek population is consistent with some (Meyre et al. 2005; Bottcher et al. 2006; Tanyolac et al. 2008), but not all (Lyon et al. 2006; Weedon et al. 2006; Meyre et al. 2007) previous replication studies (Table 4), highlighting potential population differences in the role of this haplotype on the risk of obesity and confirming the significance of conducting reproducibility studies (Li and Meyre 2013; Fatumo et al. 2022) in populations with different lifestyles, ethnic and environmental backgrounds, however functional and larger studies are needed to better understand its involvement in pathogenesis of obesity. As the K121Q amino acid change has direct functional consequences on the inhibition of the insulin pathway (Costanzo et al. 2001), it is tempting to speculate that the three variants may have cumulative, additive or synergetic effects on PC-1/ENPP1 protein function and/or expression. In line with this hypothesis, individuals with the Q–delT–G haplotype have higher serum PC-1/ENPP1 levels than the carriers of the more frequent K-insT-A haplotype (Meyre et al. 2005).

**Table 4:** The association studies of the rs1044498 (K121Q) variant and of the Q–delT–G haplotype in the *ENPP1* gene with adult obesity

Reference	Population	Sample size	Association with obesity (Yes/No)
*rs1044498 (K121Q) variant*			
Wang et al. (2011)	Europeans (meta-analysis)	24,324	Yes
Meyre et al. (2005)	French	6,147	Yes
El Achhab et al. (2009)	Moroccan	915	Yes
Peeters et al. (2009)	Belgium	1,401	No
Grarup et al. (2006)	Danish	5,961	No
Arianti et al. (2021)	Indonesian	110	No
Zhao et al. (2011)	Chinese Han	3,953	No
Hsiao and Lin (2016)	Taiwanese	1,513	No
*Q*–*delT*–*G haplotype*			
Meyre et al. (2005)	French	6,147	Yes
Tanyolac et al. (2008)	Turkey	1,553	Yes
Meyre et al. (2007)	French	5,153	No
Weedon et al. (2006)	UK	8,089	No
Lyon et al. (2006)	US & Poland	2,830	No

The association of the Q–delT–G haplotype with obesity may not be unexpected, as the 121Q obesity-risk variant is part of this haplotype. This variant has previously been shown to directly influence insulin signaling (Costanzo et al. 2001), while the other two variants in the haplotype may exert their effects by modifying gene expression, protein production, or splicing, even if their functional impact appears to be low-to-moderate based on publicly available functional prediction tools such as the RegulomeDB (https://www.regulomedb.org/regulome-search/). Despite potential functional impact of K121Q, we cannot rule out the existence of other variants with functional effects on the ENPP1 protein (Stella et al. 2016), while there is a continuous effort to highlight genetic variants associated with ENPP1 deficiency, a rare disorder characterized by pathological calcification (Mercurio et al. 2022).

We acknowledge that Q–delT–G haplotype frequency was relatively low (3.3%) in our adult Greek cohort with obesity, and moreover, lower than findings of other studies (e.g. Meyre et al. (2005) reported 11.5% in children with obesity and Tanyolaç et al. (2008) reported 14.2% in adults with obesity). This low frequency limits statistical power and increases the likelihood of Type II errors, as true associations might not be detected due to small carrier counts. Furthermore, our findings may raise concerns about reproducibility and potential Type I errors due to modest effect size estimates and thus, necessitates replication in larger cohorts.

Our study, is, to our knowledge, the first to explore the replication of association of *ENPP1* gene variants and haplotypes with risk of adult severe obesity in a population of Southern Europe with limited genetic data and high rates of obesity, as the Greek population, making thus our study novel. Studying diverse populations that may live under different environmental contexts is important in genetic studies, as it may yield novel discoveries and provide insights into the genetic basis of complex diseases such as obesity. Despite the uniform nature of the studied population, there are some limitations to consider. First, the modest sample size, compared to previous studies (Meyre et al. 2005; Tanyolac et al. 2008), makes our study underpowered, and thus more research and large-scale studies are required to identify the role of *ENPP1* gene variants with obesity. Our study’s power to detect associations for individual variants is limited by suboptimal sample size and allele frequency, particularly for variants with modest effect sizes. Consequently, some true associations may not reach statistical significance, raising the possibility of false negatives. This limitation suggests that absence of association for individual variants should be interpreted cautiously, and replication in larger, independent cohorts is warranted to confirm or refute these single-variant associations.

Importantly, our haplotype analysis results would not be significant due to stringent correction for multiple testing and thus, we cannot rule out a false positive association. Correction for multiple testing (e.g., the Bonferroni correction) was not applied due to the suboptimal size of our cohorts and our aim to replicate previous significant associations in larger cohorts. Our findings should therefore be interpreted with caution; however, such studies can provide roadmaps for future research and underscore the importance of studying diverse populations living in different environments to better understand the genetic contribution to obesity. Besides creating roadmaps, exploring multiple genetic variants across many genes in the form of polygenic risk scores (Loos 2025) would increase the clinical relevance of understanding the genetic basis of obesity in the Greek population and, theoretically, help us design personalized medicine approaches. However, larger studies are needed further to highlight the clinical relevance of our studied *ENPP1* variants and to incorporate them into polygenic risk scores.

Furthermore, while we adjusted our models for age and sex, we did not account for other factors, such as lifestyle, diet, and medication use that might influence our genetic associations. These data were unavailable at the recruitment period; however, we acknowledge their potential to modify obesity associations and emphasize the need for accounting for these factors in future studies. Adjusting our regression models for sex was an initial step in our exploratory study to account for sex differences observed between the two groups (i.e., the percentage of males was higher in the control group). We acknowledge that sex-stratified and genotype/haplotype by sex interaction analyses would provide further insights; however, this was out of the scope of the present study due to our limited statistical power. A gender difference in association studies has been observed for variants in the *ENPP1* gene. For example, in the study by Tanyolac et al. (2008), the risk haplotype showed an association with obesity in males, but not in females. Gender differences may arise from various factors, including differences in genetic background or gender-specific environmental factors.

In conclusion, we replicated the association of the haplotype Q–delT–G in the *ENPP1* gene with increased risk of severe obesity in an adult Greek population, providing, for the first time, to our knowledge, novel evidence regarding the role of *ENPP1* haplotypes in adult severe obesity susceptibility in this region of South Europe. We could not replicate the association of the three studied variants with obesity. Identifying carriers of the risk haplotype could inform personalized prevention strategies, while our results suggest the potential to integrate genetic information into risk assessment frameworks for obesity. Future research in larger cohorts and meta-analyses is needed to elucidate the role of *ENPP1* variants in the risk of obesity and to explore potential therapeutic implications of incorporating genetic information.

## Supplementary Information

Below is the link to the electronic supplementary material.

Supplementary Material 1

## Data Availability

All data can be found within the main manuscript and the supplement. Raw data can be available upon request from the corresponding author.
